# Effects of electron density force to 1.0 and fill to 1.0 on VMAT treatment plans for lung SBRT

**DOI:** 10.1002/acm2.14488

**Published:** 2024-09-03

**Authors:** Yelda Elcim

**Affiliations:** ^1^ Department of Radiation Oncology University of Health Sciences Gulhane Medical Faculty Ankara Turkey

**Keywords:** electron density (ED), lung, Monte Carlo (MC), stereotactic body radiation therapy (SBRT), volumetric modulated arc therapy (VMAT)

## Abstract

**Purpose:**

The aim of this study is to determine the effect of forcing and filling the electron density (ED) to 1.0 of the planning target volume (PTV) overdose distribution in lung SBRT treatment leading to shortening patient treatment time and increasing patient comfort by reducing MU/fraction due to ED manipulation effect.

**Methods:**

In this study, 36 lung SBRT plans of 12 suitable patients who prescribed a total dose of 50 Gy in five fractions were generated with Monaco v.5.10 TPS using the Monte Carlo (MC) algorithm and volumetric modulated arc therapy (VMAT) technique by PTV ED values forcing as well as filling to 1.0 and comparatively assessed. The first group of plans was created by using the patient's original ED, second and third groups of plans were reoptimized by forcing and filling the ED of PTV to 1.0, respectively, therefore acquiring a new dose distribution which lead to comparatively assessment the effects of changes in ED on PTV and OAR doses.

**Results:**

Assessment of treatment plans revealed that mean MU/fx numbers were decreased by 76% and 75.25% between Groups 1 and 2, Groups 1 and 3, respectively. The number of segments was also reduced in Group 1 by up to 15% compared with Groups 2 and 3. Maximum HI and CI differences for PTV between Groups 1 and 2 were less than 1% and Groups 1 and 3 were 1.5% which indicates all 3 group plans were comparable in terms of dose distribution within PTV.

**Conclusions:**

Forcing and filling the ED of PTV to 1.0 strategy has provided reduced a number of segments and MU/fx without a significant change in PTV mean and maximum doses, thereby decreasing treatment time and patient discomfort during treatment. This process should be considered in line of a potential number of patients as well as prescribed dose and MU/fx numbers.

## 1 INTRODUCTION

Stereotactic body radiation therapy (SBRT) has emerged as a game‐changer in the treatment ofperable early‐stage lung cancer. Its precise targeting allows for high doses of radiation to be delivered to the tumor while minimizing exposure to surrounding healthy tissues. This has significantly improved outcomes forare not candidates for surgery due to medical reasons. However, its effectiveness in patients medically suitable for surgery is unclear.^1^ The American Cancer Society's estimates for lung cancer in the United States for 2022; Lung cancer is by far the leading cause of cancer death, making up almost 25% of all cancer deaths. Each year, more people die of lung cancer than of colon, breast, and prostate cancers combined. SBRT is one such technique that has shown efficacy as an upfront treatment for lung cancer.^2^ For lung cancer, the radiation is usually in the form of x‐ray beams that come from a machine outside the body. Conventionally, lung cancer SBRT has been delivered using three‐dimensional (3D) non‐coplanar beams^3^ or IMRT. Recently, volumetric modulated arc therapy (VMAT) was introduced to treat various disease sites, including lung SBRT.^4^

SBRT was derived from intracranial stereotactic radiosurgery (SRS), which was first presented in 1951.^5^ In 2001, SRS was approved by the US Food and Drug Administration to treat areas throughout the body and the first SBRT delivery system was established. Immobilization devices and improved real‐time imaging have allowed clinicians to administer high ablative doses to accurately target the tumor.^6^

The main aim of motion management in SBRT, especially in lung cases, is to reduce CTV and planning target volume (PTV) margins, therefore decreasing critical organ doses while escalating GTV dose by relatively immobilizing the target volume.

Retrospective calculation using MC‐based algorithms in patients enrolled in Radiation Therapy Oncology Group (RTOG) 0915 for the treatment of peripheral non‐small cell lung cancer (NSCLC) lesions indicated that only 25% actually met the RTOG dosimetric criteria, including conformity index and ratio of 50% isodose volume to PTV.^7^ Literature on the correlation of Monte Carlo (MC) based dose distributions and outcome, in the context of SBRT for treatment of lung cancer, is limited.^8^

The presence of inhomogeneous media can also affect dose calculation accuracy. Several studies have examined the impact of different dose calculation algorithms on the dose delivered to inhomogeneous media, particularly in the lungs.^9^, ^10^, ^11^

Respiratory tumor motion increases the position uncertainty of the target and normal tissues in NSCLC, and achieving local tumor control requires understanding and incorporating tumor motion into the simulation, planning, and delivery of radiotherapy (RT), leading to multiple opportunities to monitor and mitigate motion during simulation and treatment.^12^ Breath‐holding techniques usually require the patient to spend more time, which can go as high as 45 min depending on the patient's performance, in the treatment room. The longer the duration of treatment, the more tired the patient becomes, and patient comfort deteriorates. Therefore, higher treatment duration may compromise treatment accuracy and reproducibility. Higher treatment time may compromise the treatment accuracy and repeatability.

The technologies covered by this report are motion‐encompassing methods, respiratory gated techniques, breath‐hold techniques, forced shallow‐breathing methods, and respiration‐synchronized techniques. Respiratory motion affects all tumor sites in the thorax and abdomen, though the disease of most prevalence and relevance for radiotherapy is lung cancer.^13^

Tumor tracking and breathing‐adapted radiotherapy techniques allow for the management of respiratory motion to achieve optimal patient treatment.^14^ The effect of PTV target dose distribution and critical organ doses was investigated by forcing the electron density (ED) to 1 within the PTV contour. CTV/PTV was created by giving small margins to GTV.^15^, ^16^, ^17^ Since this group of patients was treated by a breath‐holding technique during treatment, MU/fx was evaluated comparatively to investigate whether forcing the ED to 1.0 was affected or not. ED changes are high in lung radiotherapy due to the highly heterogeneous structure of the treatment site. X‐rays will act as the primary attenuation in dose calculations in lung tumors and secondary electrons will accumulate in neighboring environments such as the spinal cord or chest wall. Therefore, it is very important to make correct dose optimization in treatment planning.

Monaco v5.10 based on the MC algorithm, which is accepted as the gold standard,^18^ was used in dose calculations in TPS. Electron densities within the lung structure are less than 1.0. However, tumor structures in the lung may correspond to smaller than 1 and different densities.

In the Monaco treatment planning system, there are forces to ED and fill to ED parameters that the user can change while calculating the dose with the MC algorithm. With the force to 1.0 ED parameter, different electron densities in the structure are converted to 1.0 and used in dose calculation. With the fill to 1.0 ED parameter, it takes electron densities less than 1 in the structure to 1, while electron densities greater than 1.0 in the structure are taken into account in the dose calculation without changing them.

## 2 METHODS

Patients referred to our department after a thorough multidisciplinary assessment for the management of pulmonary metastases by SBRT were included in the study. A total dose of 50 Gy in five fractions was given to 12 cases with either lung metastases or inoperable early‐stage primary lung tumor patients. Treatment plans of all patients were optimized to provide 50 Gy dose to 95% isodose line of PTV while limiting organs at risk (OAR) such as proximal tracheobronchial tree, left lung, right lung, heart, great vessels, esophagus, spinal cord, chest wall, brachial plexus and rib dose limitations as per the QUANTEC refer to. Written informed consents of all patients were obtained prior to consolidative thoracic RT with institutional tumor board approval at our tertiary cancer center, and this study has been conducted in compliance with the Declaration of Helsinki principles and its later amendments.

In this study, computed tomography (CT) images of 2 mm slice thickness were acquired on the Toshiba CT, PTV volumes were contoured with the Monaco contouring system with a 3 mm margin to the GTV. Treatment plans were also generated with Monaco v.5.10 TPS using the MC algorithm and VMAT technique. VMAT treatment plans were generated with single beam and double rotation arc therapy with 6 MV photon energy on Elekta Infinity linear accelerator. The patients’ tumor movement was managed during treatment using the Elekta Active Breathing Coordinator™ (ABC) system by moderate DIBH (deep inspiration breath‐hold) technique.

Three plans were generated for each patient. The first reference group (Group 1) of plans was generated by using the patient's original ED. The second group of plans was generated by reoptimizing PTV by forcing the ED to 1.0 leading to a new dose distribution (reoptimized). The third group of plans was generated by the three groups of plans that were comparatively assessed for the effect of changes in ED on PTV and OAR doses. Calculation parameters for the MC algorithm in Monaco TPS Grid spacing are 3 mm, Statistical uncertainty 1%, maximum control point 30 per beam, and minimum segment width 5 mm.

The mean and maximum doses for PTV; HI (Homogeneity Index), CI (Conformity Index), V20 (Volume which receives 20 Gy dose), and MLD (Mean Lung Dose) as well as MU/fx (Monitor Unit per fraction) values were obtained from each patient's DVH and plan data which were compared for three groups.

Table 1 shows each patient PTV volume as well as minimum, maximum, and mean values of HU/ED for each PTV and Table 2 shows MU/fx values and number of segments for each plan group.

**TABLE 1:** Minimum, maximum, and mean values of HU/ED.

		PTV (HU/ED)		
Patient	PTV (cc)	Minimum	Maximum	Mean
1	7.692	−850/0.163	15/1.012	−712/0.304
2	7.684	−1000/0.001	39/1.027	−502/0.510
3	2.402	−909/0.100	28/1.020	−689/0.324
4	9.117	−996/0.007	668/1.385	−255/0.746
5	4.059	−932/0.075	29/1.021	−619/0.393
6	19.852	−846/0.167	1189/1.644	−119/0.869
7	21.56	−1002/0.001	263/1.164	−145/0.850
8	3.778	−1007/0.001	814/1.456	−758/0.242
9	13.	−1003/0.001	629/1.366	−453/0.558
10	7.059	−985/0.019	165/1.104	−501/0.512
11	6.044	−996/0.007	48/1.032	−638/0.373
12	9.182	−998/0.007	668/ 1.438	−229/0.864

Abbreviations: ED, electron density; PTV, planning target volume.

**TABLE 2:** MU/fx values and number of segments for each plan groups.

	Group 1		Group 2		Group 3	
Patient	MU/fx	Segment	MU/fx	Segment	MU/fx	Segment
1	2965	230	1989	334	2017	336
2	5098	271	3474	265	3649	258
3	6867	289	4696	317	4490	290
4	4074	329	2649	326	2647	325
5	3736	331	2949	331	2768	324
6	3075	275	2714	284	2647	273
7	2901	331	2327	334	2370	335
8	7531	307	3752	260	4777	287
9	2498	336	2355	331	2166	329
10	3601	324	2614	293	2250	281
11	3285	288	3740	290	3826	286
12	3926	330	2750	328	2648	324

Both patient and QA plans were statistically evaluated using the IBM SPSS Statistics Version 26.0.0.0 program. To confirm the patient QA plans’ dosimetric precision, each plan was recalculated in accordance with AAPM TG‐218,^19^ which requires 3% within 3 mm gamma index accuracy, and analyzed by OmniPro‐IMRT with MatriXX (IBA dosimetry, Schwarzenbruck, Germany). Moreover, the differences found between gamma indexes in these patient QA's were not statistically significant (*p* > 0.05).

## 3 RESULTS

**FIGURE 1:**
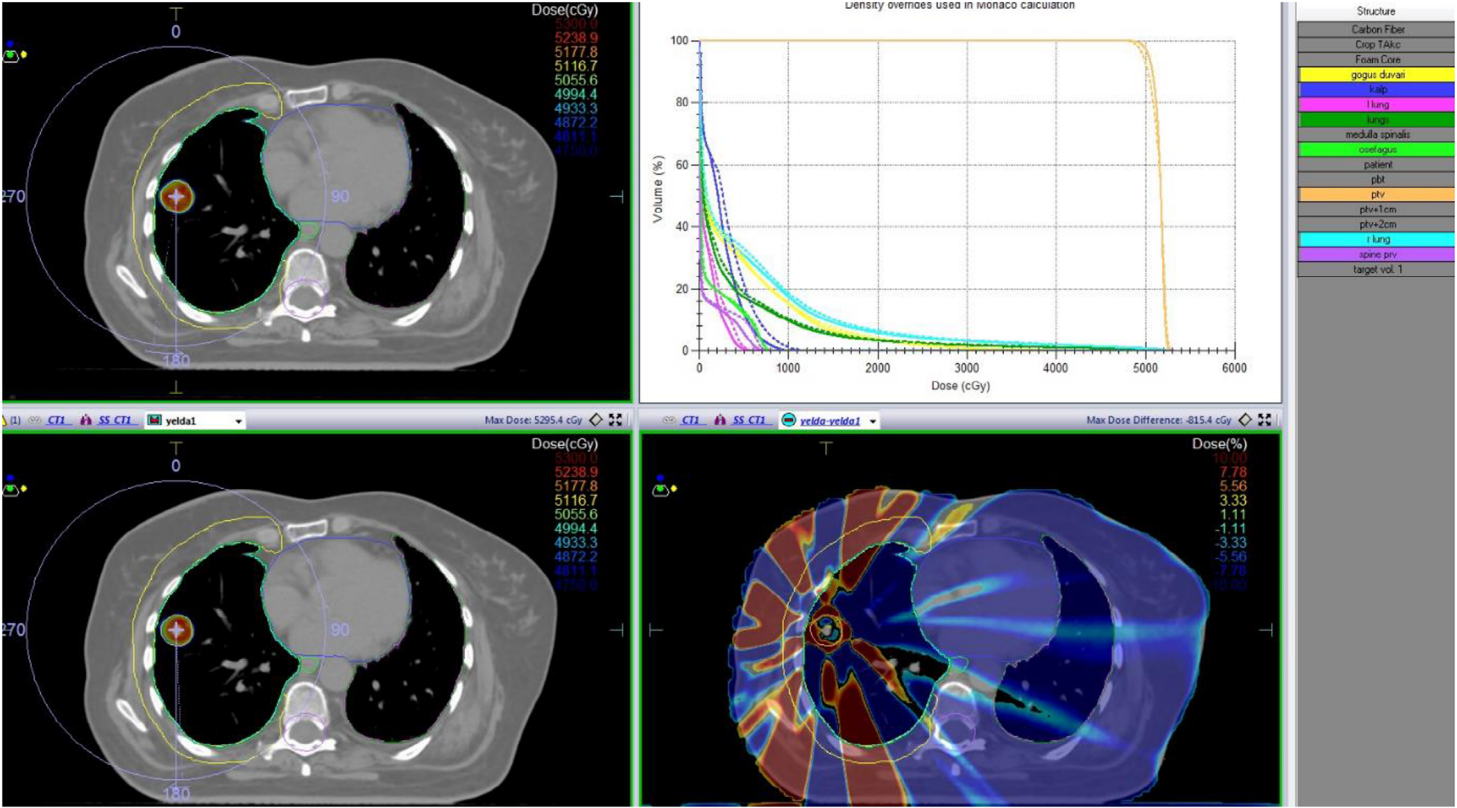
A representative example for comparison of Groups 1 and 2.

**FIGURE 2:**
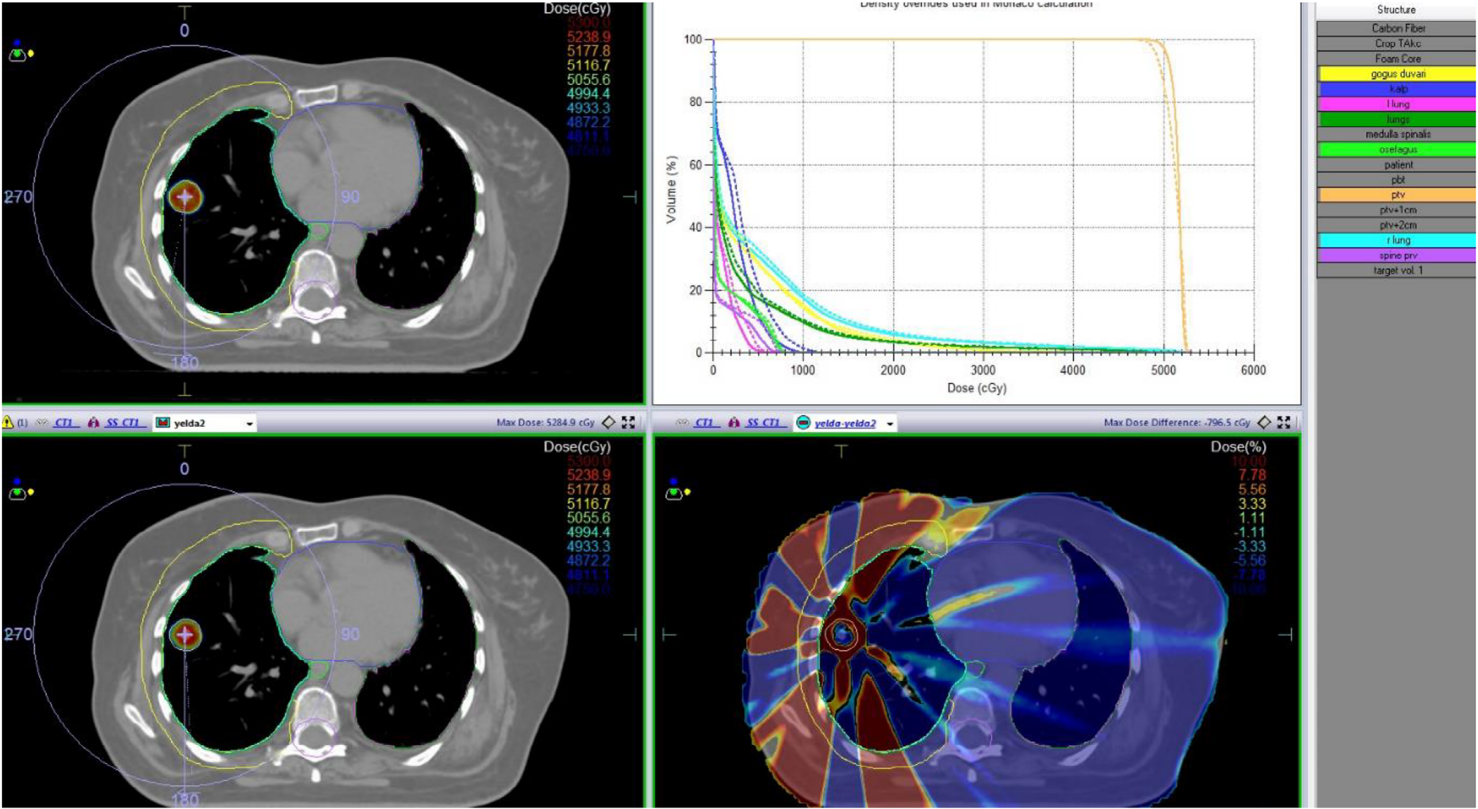
A representative example for comparison of Groups 1 and 3.

PTV dose distribution assessment between treatment plans using Anova analysis revealed that there was no statistical significance in terms of PTV mean and maximum doses (*p* > 0.05). Figures 1 and 2 show comparative DVH graphics and dose distributions between Groups 1 and 2 and Groups 1 and 3, respectively.

Analyzing V20 and MLD for ipsilateral lung using the Friedman test showed no statistical difference (*p* > 0.05). Table 2 shows MU/fx values and the number of segments for each plan group.

Assessment of treatment plans revealed that plan MU/fx numbers were decreased by a mean of 76% between Groups 1 and 2 and by 75.25% between Groups 1 and 3. The number of segments was also reduced in Group 1 up to 15% compared with Groups 2 and 3.

Figure 2 shows MU/fx distributions among the groups of plans for each patient. For all patients except for patient number 4, force to 1.0 (Group 2) and fill to 1.0 (Group 3) resulted in reduced MU/fx.

**FIGURE 3:**
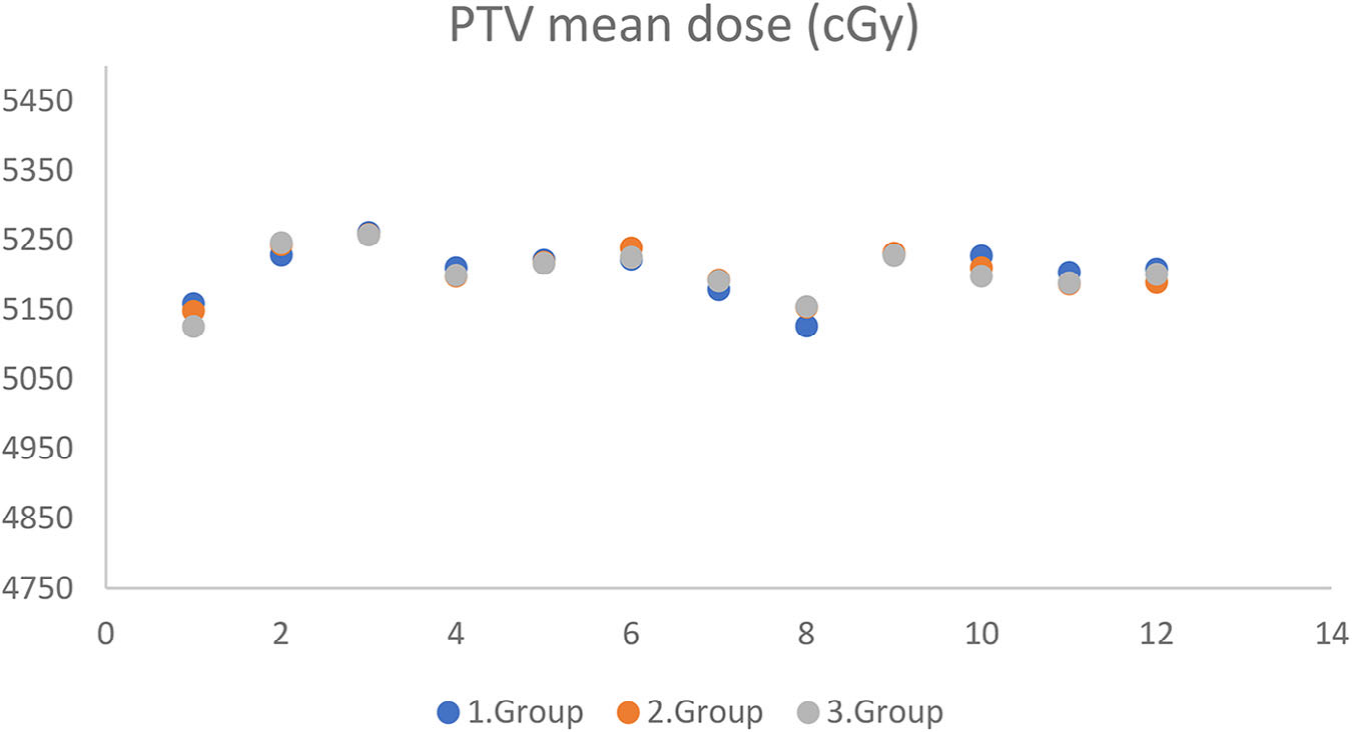
Planning target volume (PTV) mean dose values for each group.

**FIGURE 4:**
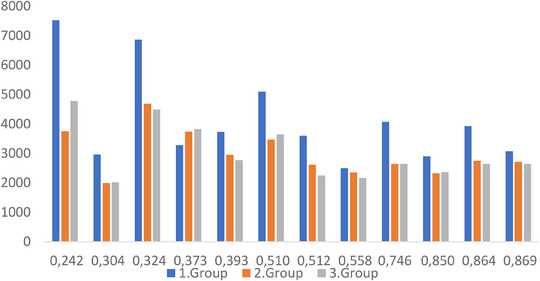
Each patient's MU/fx distributions among the groups.

Maximum HI and CI differences for PTV between each group were less than 1% and 1.5%, respectively, which indicates all 3 group plans were comparable. Figure 3 shows PTV mean dose values for each group and Figure 4 shows Each patient's MU/fx distributions among the groups.

## 4 DISCUSSION

In this study, we created 3 SBRT lung plans for each patient with the aim of 95% prescribed dose to cover 100% of PTV volume which has three different ED values; original ED, force ED, and fill ED.

Plans were reoptimized in both planning systems using the same optimization objectives, and normalized so the prescription dose covers 95% of the PTV. MUs required to deliver each plan were compared. Forcing either the ITV or PTV electron density has a significant effect on the optimization and MUs of 4D lung SBRT plans, especially for PTV density overrides. The effect is more pronounced in Monaco/XVMC versus Eclipse /AcurosXB. The reduction is not found to be linked to the ITV‐to‐GTV or PTV‐to‐GTV ratio. Distortion and artifacts were caused by respiratory movement which were minimized by BreathHoldCT. More solid and clear boundary images could be obtained with BreathHoldCT. ED and Hounsfield Unit values could be calculated more accurately, independent of respiratory motion with BreathHoldCT. Lung density is increasing in NormalCT and AverageCT, which affects the dose distribution in healthy lungs.^20^

Low‐density PTV overrides improved the plan quality and accuracy for tumor diameters less than 22 mm only. Although an ITV override generated the most significant increase in accuracy, the low‐density PTV plans had the additional benefit of plan quality improvement. Although this study and others agreed that density overrides improve the treatment of SBRT, the optimal density override and the conditions under which it should be applied were found to be department‐specific, due to variations in commissioning and calculation methods.^21^

## 5 CONCLUSION

Breath‐holding techniques are utilized for respiratory motion management in pulmonary SBRT applications. While breath‐holding techniques are effective for limiting tumor motion, the use of these techniques typically prolongs the time required to deliver treatment fractions since the beam is in a predetermined phase of the breathing cycle. Prolonged duration of treatment may be burdensome for patients under robust immobilization and may lead to inaccuracies in treatment delivery. In this context, shortening the time to deliver the SBRT fractions may have clinical implications from the perspective of patient compliance and treatment accuracy.

This study shows that forcing and filling the ED to 1.0 improves the SBRT lung treatment plan and it is seen that the treatment has a great contribution to reducing the treatment time of the patient by reducing the number of MU/fx.

Clearly, more work is needed to evaluate the effect of ED force to 1.0 or fill to 1.0 in dose calculation parameters for lung SBRT management. By forcing and filling the ED of the PTV to 1.0, the MU/fx can be reduced, thereby reducing the length of time the patient is in the treatment room, thereby improving patient comfort during treatment. This process should be considered and investigated together with clinical patient potential, prescribed dose, MU/fx, PTV volume (cc), and even PTV number.

## AUTHOR CONTRIBUTIONS

The corresponding author provided study design, data collection and analysis, manuscript preparation, and its final review.

## CONFLICT OF INTEREST STATEMENT

The author declares no conflicts of interest.
